# Recognition of Multiple Imbalanced Cancer Types Based on DNA Microarray Data Using Ensemble Classifiers

**DOI:** 10.1155/2013/239628

**Published:** 2013-08-26

**Authors:** Hualong Yu, Shufang Hong, Xibei Yang, Jun Ni, Yuanyuan Dan, Bin Qin

**Affiliations:** ^1^School of Computer Science and Engineering, Jiangsu University of Science and Technology, No. 2 Mengxi Road, Zhenjiang 212003, China; ^2^Department of Radiology, Carver College of Medicine, The University of Iowa, Iowa City, IA 52242, USA; ^3^School of Biology and Chemical Engineering, Jiangsu University of Science and Technology, No. 2 Mengxi Road, Zhenjiang 212003, China

## Abstract

DNA microarray technology can measure the activities of tens of thousands of genes simultaneously, which provides an efficient way to diagnose cancer at the molecular level. Although this strategy has attracted significant research attention, most studies neglect an important problem, namely, that most DNA microarray datasets are skewed, which causes traditional learning algorithms to produce inaccurate results. Some studies have considered this problem, yet they merely focus on binary-class problem. In this paper, we dealt with multiclass imbalanced classification problem, as encountered in cancer DNA microarray, by using ensemble learning. We utilized one-against-all coding strategy to transform multiclass to multiple binary classes, each of them carrying out feature subspace, which is an evolving version of random subspace that generates multiple diverse training subsets. Next, we introduced one of two different correction technologies, namely, decision threshold adjustment or random undersampling, into each training subset to alleviate the damage of class imbalance. Specifically, support vector machine was used as base classifier, and a novel voting rule called counter voting was presented for making a final decision. Experimental results on eight skewed multiclass cancer microarray datasets indicate that unlike many traditional classification approaches, our methods are insensitive to class imbalance.

## 1. Introduction

Microarray technology allows large-scale and parallel measurements for expression of around thousands, perhaps even tens of thousands, of genes. It has been one of the most successful molecular biology technologies in the postgenome era and has been widely applied to predict gene functions [1], provide invaluable information for drug discovery [2, 3], investigate gene regulatory mechanisms [4, 5], find new subtypes of a specific tumor [6, 7], and classify cancers [8, 9]. Among these applications, cancer classification, which has been the subject of extensive research all around the world, is most promising [10]. However, microarray data are known to have some features, such as high dimension, small sample, high noise, high redundancy, and skewed class distribution which is called class imbalance problem. Class imbalance occurs when examples from one class outnumber those of the other class, which results in great underestimation of the classification performance of the minority, thereby further affecting the evaluation precision of the overall classification performance. In other words, developing a clinical tumor diagnostic system is meaningless if class imbalance is not considered.

Recent studies have addressed this problem in the context of cancer classification based on microarray data [11–18]. Unfortunately, most existing work has only considered binary-class imbalance and ignored the multiclass problem, that is, identifying multiple imbalanced tumor types or several skewed subtypes of a special tumor. Applying traditional supervised learning algorithms that solve minimum classification errors will provide inaccurate classification results. Furthermore, addressing skewed multiclass problems is more difficult than dealing with binary-class imbalance problems [19].

Generally speaking, support vector machine (SVM) is the best choice for classifying cancer microarray data because of its advantages, such as its high generalization capability, absence of local minima, and adaptability for high-dimension and small sample data [20]. However, SVM was initially designed for binary-class problems. Therefore, to apply SVM to multiclass problems, it should be reconfigured for multiple binary-class problems by using a coding strategy [21]. Previous studies have presented several well-known coding strategies, including one-against-all (OAA), one-against-one (OAO), decision directed acyclic graph (DDAG), and error correcting output codes (ECOC). These strategies have also been used to classify multiclass cancer microarray data [22–24]. Statnikov et al. [25] systematically assessed these strategies by performing experiments and found that OAA often produces better classification accuracy. In the present study, we use OAA as a baseline coding strategy. We also note that this decomposition can further damage the equilibrium of training instances. Therefore, one approach for effective class imbalance correction should be carried out in each binary-class branch.

In this paper, we attempted to address the multiclass imbalance classification problem of cancer microarray data by using ensemble learning. Ensemble learning has been used to improve the accuracy of feature gene selection [26] and cancer classification [27–29]. First, our method used OAA coding to divide multiclass problems into multiple binary-class problems. Next, we designed an improved random subspace generation approach called feature subspace (FSS) to produce a large number of accurate and diverse training subsets. We then introduced one of two correction technologies, namely, either decision threshold adjustment (THR) [17] or random undersampling (RUS) [30], into each training subset to deal with class imbalance. Finally, a novel voting rule based on counter voting was presented, which made the final decision in ensemble learning. We evaluated the proposed method by using eight multiclass cancer DNA microarray datasets that have different numbers of classes, genes, and samples, as well as class imbalance ratios. The experimental results demonstrated that the proposed method outperforms many traditional classification approaches because it produces more balanced and robust classification results.

The rest of this paper is organized as follows. In Section 2, the methods referred to in this study are introduced in detail.  Section 3 briefly describes the datasets that were used.  Section 4 introduces performance evaluation metrics and experimental settings. Results and discussions are presented in Section 5.  Section 6 summarizes the main contributions of this paper.

## 2. Methods

### 2.1. Coding Strategies for Transforming Multiclass into Multiple Binary Classes

Coding strategies are often used to transform multiclass into multiple binary-classes [21]. OAA, OAO, and ECOC can be described by a code matrix *M*, where each row contains a code word assigned to each class, and each column defines a binary partition of *C* classes. Specifically, we assign +1, −1, or 0 for each element in *M*. An element *m*_*ij*_ with +1 value indicates that the *i*th class is labeled as positive for the *j*th binary classifier, −1 represents that the *i*th class in the *j*th binary classifier is labeled as negative, and 0 means that the *i*th class does not participate in the induction of the *j*th classifier.

**Figure 1:**
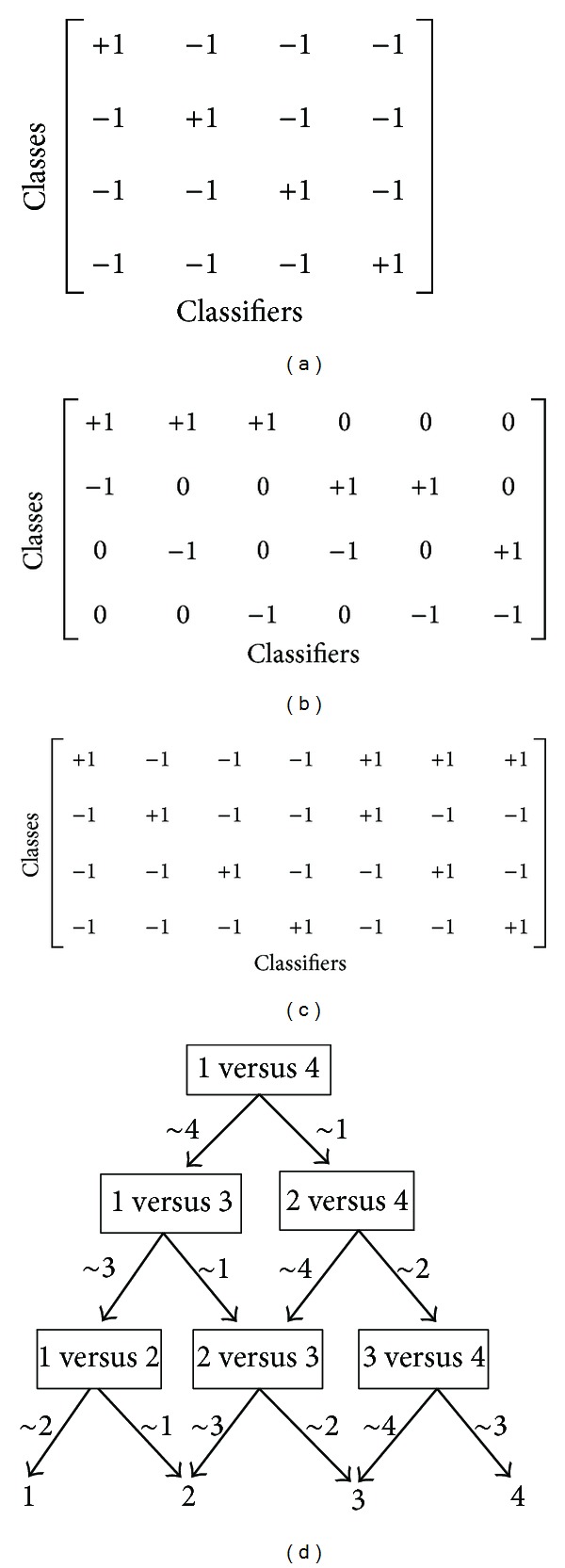
Code matrices of different coding strategies for a classification problem with four classes, where (a) is OAA coding strategy, (b) is OAO coding strategy, (c) is ECOC coding strategy, and (d) is DDAG decomposition strategy.

Without loss of generality, a problem of four classes is assumed; that is, *C* = 4. OAA generates *C* classifiers in which each one is trained to distinguish a class from the remaining classes. The code matrix of OAA is presented in Figure 1(a). In practical applications, OAA assigns the class label with the highest decision output value to the test instance. Unlike OAA, OAO trains *C* × (*C* − 1)/2 binary classifiers and assigns each one by using only two original classes and simply ignoring the others. Its code matrix is shown in Figure 1(b). The decoding rule of OAO is majority voting; that is, the test instance is designated to the class with the most votes. ECOC proposed by Dietterich and Bariki [31] uses error correcting codes to denote *C* classes of a multiclass problem. For each column of the code matrix, one or several classes are denoted as positive, and the remainder is designated as negative. In ECOC, hamming distance is applied as decoding strategy. In particular, when using an exhaustive code to construct the code matrix of ECOC, it can generate more binary classifiers than OAA and OAO. The size of ECOC is 2^*C*−1^ − 1, and its code matrix is described in Figure 1(c).

DDAG [32] has the same coding rule as OAO but uses a totally different decoding strategy. It organizes all binary classifiers into one hierarchical structure (see Figure 1(d)) and makes a decision for test samples from root to leaf, which is helpful for decreasing time complexity of the testing process.

To our knowledge, no previous work has considered the effect of class imbalance on these coding strategies, although some have indicated that it is, in fact, harmful [33, 34]. In this paper, we proposed two solutions for this problem and used OAA coding as the baseline.

### 2.2. Feature Subspace Generation Technology

The performance of ensemble learning is related to two factors: accuracy and diversity of base classifiers [35]. The generalization error of ensemble learning *E* can be calculated by using the following equation:

(1) $$\begin{array}{c} E=\bar{E}-\bar{A}, \end{array}$$

where $\bar{E}$ and $\bar{A}$ are averages of generalization errors and diversities, respectively. Therefore, to create a successful ensemble learning model, two factors should be considered simultaneously. The more accurate each base classifier and the more diverse different base classifiers, the better the classification performance of the ensemble learning. However, these two factors are conflicting; that is, with the increase in average accuracy, the average diversity inevitably declines, and vice versa. Many effective ensemble learning methods are available, including Bagging [36], AdaBoost [37], random subspace [38] and random forest [39]. However, we have observed that these methods are not sufficiently effective in classifying high-dimensional data. Therefore, we used a modified random subspace method [38], and proposed an FSS generation strategy, which is described below.

DNA microarray data are known to contain numerous noisy and redundant genes, which can negatively affect classification performance and should thus be preliminarily eliminated. FSS generation strategy uses hierarchical clustering, which uses Pearson correlation coefficient (PCC) as a similarity measure to delete redundant genes and signal-to-noise ratio (SNR) feature selection method [6] to remove noisy genes. PCC evaluates the similarity between two genes *g*_*i*_ and *g*_*j*_ by using the following equation:

(2) $$\begin{array}{c} \text{PCC}\left(g_{i},g_{j}\right)=\frac{\sum_{k=1}^{m}\left(g_{ik}-\bar{g_{i}}\right)\left(g_{jk}-\bar{g_{j}}\right)}{\sqrt{\sum_{k=1}^{m}{\left(g_{ik}-\bar{g_{i}}\right)}^{2}}\sqrt{\sum_{k=1}^{m}{\left(g_{jk}-\bar{g_{j}}\right)}^{2}}}, \end{array}$$

where *g*_*ik*_ is the expression value of the gene *g*_*i*_ on the *k*th sample, $\bar{g_{i}}$ represents the mean value of *g*_*i*_, and *m* denotes the number of training samples. A larger PCC between two genes indicates that the genes have greater similarity. Using this method ensures that all genes could be grouped into *K* clusters, where *K* is the number of clusters. Obviously, redundant genes can emerge in the same clusters. For this, we use the SNR feature selection method [6] to select differentially expressed genes in each cluster, with the computational formula listed as follows:

(3) $$\begin{array}{c} \text{SNR}\left(x_{i}\right)=\frac{\left|\mu_{+}-\mu_{-}\right|}{\left(\sigma_{+}+\sigma_{-}\right)}, \end{array}$$

where *μ*_+_ and *μ*_−_ are mean values of gene *g*_*i*_ in positive class and negative class, and *σ*_+_ and *σ*_−_ are their standard deviations, respectively. The extracted features are clearly closely correlated with the classification task without being redundant with each other. We call the space that merely contains the *K* extracted genes the feature space from which multiple feature subspaces can be generated. If the dimension of feature subspace is *D*, where *D* ≤ *K*, then a feature subspace can be generated by using the following random project function:

(4) $$\begin{array}{c} P\left(R^{K}\right)\in R^{D}. \end{array}$$

By using the random project function *P*, we can repeatedly produce multiple diverse feature subspaces. For a given high-dimensional training set *T*, the pseudocode description of the FSS generalization algorithm is presented in Pseudocode 1.

 We also analyze the reason behind the ability of FSS to promote equilibrium relationship between accuracy and diversity of base classifiers. Suppose *f* is one gene in feature space that has been integrated into feature subspace FSS_*i*_. Then the probability that *f* has simultaneously appeared in another feature subspace FSS_*j*_ is

(5) $$\begin{array}{c} P\left(f\in {\text{FSS}}_{j}\;|\;f\in {\text{FSS}}_{i}\right)=\frac{D}{K}. \end{array}$$

This equation means that for any two feature subspaces, their coselection rate is, in theory, about *D*/*K*. Moreover, because any two genes in the feature space can be regarded as approximatively nonredundant, the theoretical diversity between two feature subspaces div can be computed by the following:

(6) $$\begin{array}{c} \mathrm{div}=\frac{\left(K-D\right)}{K}. \end{array}$$

When *K* is much larger than *D*, diversity among the feature subspaces can be guaranteed. *D* is an important parameter that influences the accuracy of base classifiers and should not be assigned an overly small value. In addition, a constructed ensemble learning model theoretically has *C*_*K*_^*D*^ different combinations, such that the number of different combinations is deduced to reach its peak value when *D* = *K*/2.

### 2.3. Support Vector Machine and Its Correction Technologies for Class Imbalance Problem

SVM, which is based on the structural risk minimization theory, is one of the most popular classification algorithms. The decision function of SVM is listed as follows:

(7) $$\begin{array}{c} h\left(x\right)=\mathrm{sgn}\left(\sum_{i=1}^{\text{sv}}\alpha_{i}y_{i}K\left(x,x_{i}\right)+b\right), \end{array}$$

where sv represents the number of support vectors, *α*_*i*_ is the Lagrange multiplier, *b* is the bias of optimum classification hyperplane, and *K*(*x*, *x*_*i*_) denotes the kernel function. Some previous studies have found that the radial basis kernel function (RBF) generally produces better classification accuracy than many other kernel functions [20, 30]. RBF kernel is presented as

(8) $$\begin{array}{c} K\left(x_{i},x_{j}\right)=\exp\left\{-\frac{{\left|x_{i}-x_{j}\right|}^{2}}{2\sigma^{2}}\right\}, \end{array}$$

where *σ* is the parameter that indicates the width of the RBF kernel.

**Figure 2:**
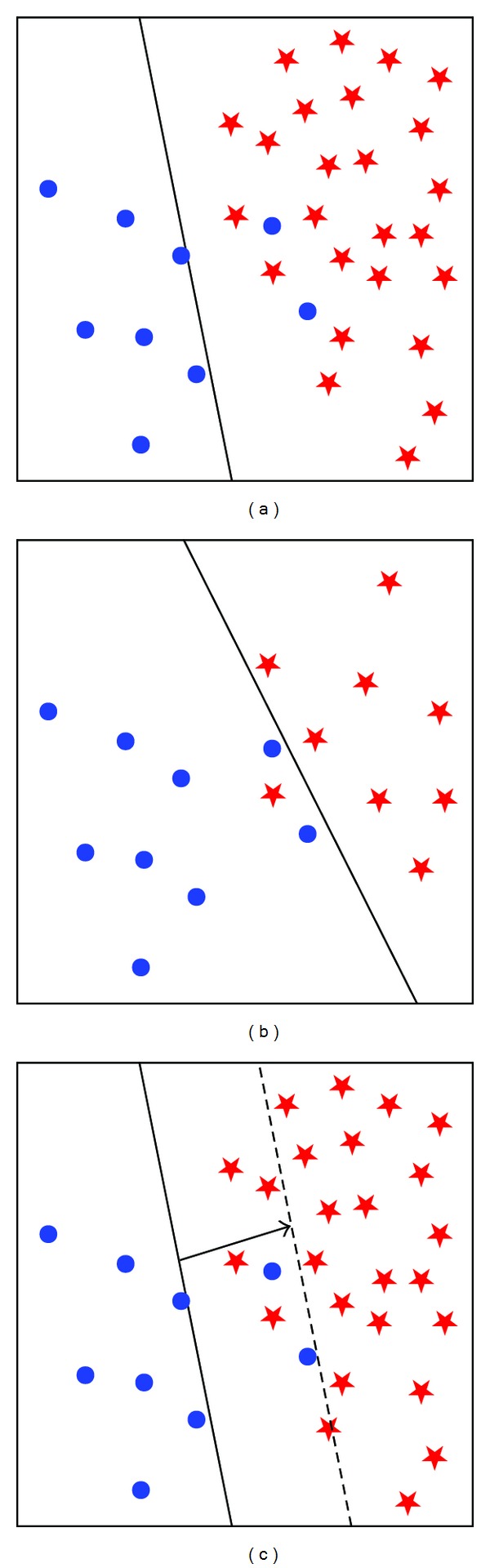
Graphical representations of original SVM and SVMs based on two different correction technologies for class imbalance problem, where (a) is original SVM modeling, (b) is SVM-RUS modeling, (c) is SVM-THR modeling. The circle points denote positive samples and the asterisk points represent negative examples, respectively.

 Although SVM is more robust to class imbalance than many other machine learning methods because its classification hyperplane only associates with a few support vectors, it can still be, more or less, affected by skewed class distribution. Previous studies [40, 41] have found that the classification hyperplane can be pushed toward the minority class if the classification data is skewed (see Figure 2(a)). 

Class imbalance correction technologies of SVM can be roughly divided into three categories: sampling [30, 40], weighting [41, 42], and decision threshold adjustment [17], that is, threshold moving. Sampling is the most direct solution for class imbalance. It increases instances of minority class [40] or decreases examples of majority class [30] to mediate the skewed scaling relation. The former is called oversampling and the latter is called undersampling. Weighting [41], which is also known as cost-sensitive learning, assigns different penalty factors for the samples of positive and negative classes. Generally speaking, the penalty factor of positive class *C*_+_ is much larger than that of negative class *C*_−_. Phoungphol et al. [42] used ramp loss function to construct a more robust and cost-sensitive support vector machine (Ramp-MCSVM) and used it to classify multiclass imbalanced biomedical data. Decision threshold adjustment based on support vector machine (SVM-THR) directly pushes classification hyperplane toward the majority class. Lin and Chen [17] suggested adopting SVM-THR to classify severely imbalanced bioinformatics data. 

In this paper, to reduce time complexity, we used SVM based on random undersampling (SVM-RUS) [30] (see Figure 2(b)) and SVM with decision threshold adjustment (SVM-THR) [17] (see Figure 2(c)) to deal with class imbalance problem. The decision threshold is adjusted by using the following default equation [17]:

(9) $$\begin{array}{c} \theta =\frac{m_{+}-m_{-}}{m_{+}+m_{-}+2}, \end{array}$$

where *m*_+_ and *m*_−_ are the number of examples that belong to the positive class and the negative class, respectively. For one test sample *x*_*i*_, supposing that the original decision function is *h*(*x*_*i*_), the adjusted decision function can be represented as *h*′(*x*_*i*_) = *h*(*x*_*i*_) − *θ*.

### 2.4. Ensemble Learning Framework Based on Feature Subspace and Counter Voting Integration Rule for Classifying Imbalanced Multiclass Cancer Microarray Data

Ensemble learning often provides a framework to generate multiple weak classifiers and aggregates these by using an integration rule to become a strong classifier. The integration rules mainly include majority voting and weighted voting. With the characteristics of multiclass problem taken into consideration and referring to the idea of majority voting, we propose a novel integration rule called counter voting. For each decomposed binary-class branch in OAA, one counter is assigned, which indicates the proportion of test sample *x*′ that belongs to the corresponding positive class. All counters compete with each other to select the category of the test sample by using the following equation:

(10) $$\begin{array}{c} h\left(x^{\prime }\right)=\underset{i\in \left\{1,2,\ldots ,C\right\}}{\arg\;\max}\left(\text{Counte}{\text{r}}_{i}\left(x^{\prime }\right)\right). \end{array}$$

**Figure 3:**
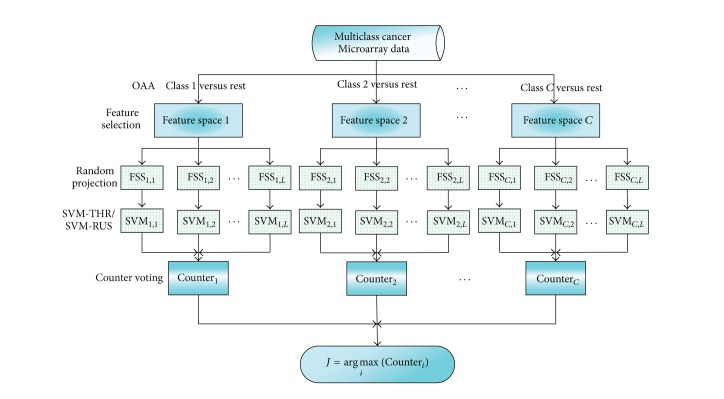
The frame diagram of the ensemble learning algorithms based on feature subspace and counter voting rule for classifying imbalanced multiclass cancer microarray data.

The pseudo-code description and graphical representation of our proposed ensemble learning algorithms are given in Pseudocode 2 and Figure 3, respectively. We call these algorithms as EnSVM-OAA(THR) and EnSVM-OAA(RUS). Figure 3 shows that if one classification task is binary, counter voting turns into majority voting. Counter voting, rather than majority voting or weighted voting, is used to classify multiclass data because generating feature space on each binary-class is more accurate than directly generating feature space on multiple classes. Our proposed ensemble learning framework also has the same time complexity as aggregating *L* SVM-OAAs by using majority voting.

## 3. Datasets

Eight skewed multiclass cancer microarray datasets [6, 7, 43–48] were used to verify the effect of our proposed ensemble learning methods, which have 3 to 26 classes, 50 to 308 instances, 2308 to 15009 genes, and imbalance ratios in the range of 2.14 to 23.17. These datasets are available at http://www.gems-system.org/, and detailed information about these data is shown in Table 1.

## 4. Performance Evaluation Metrics and Experimental Settings

When one classification task is skewed, the overall classification accuracy Acc is no longer an appropriate evaluation metric for estimating the quality of a classifier. In this case, a confusion matrix described in Table 2 is usually employed.

The description in Table 2 gives four baseline statistical components, where TP and FN denote the number of positive examples which are accurately and falsely predicted, respectively, and TN and FP represent the number of negative samples that are predicted accurately and wrongly, respectively. Two frequently used measures for class imbalance problem, namely, *F*-measure and *G*-mean, can be regarded as functions of these four statistical components and are calculated as follows:

(11) $$\begin{array}{c} F\text{-measure}=\frac{2\times \text{Precision}\times \text{Recall}}{\text{Precision}+\text{Recall}},\\ G\text{-mean}=\sqrt{\text{TPR}\times \text{TNR}}, \end{array}$$

where Precision, Recall, TPR, and TNR can be further defined as follows:

(12) $$\begin{array}{c} \text{Precision}=\frac{\text{TP}}{\text{TP}+\text{FP}},\\ \text{Recall}=\text{TPR}=\frac{\text{TP}}{\text{TP}+\text{FN}},\\ \text{TNR}=\frac{\text{TN}}{\text{TN}+\text{FP}}. \end{array}$$

The overall classification accuracy Acc can be calculated by using the following equation:

(13) $$\begin{array}{c} \text{Acc}=\frac{\text{TP}+\text{TN}}{\text{TP}+\text{TN}+\text{FP}+\text{FN}}. \end{array}$$

However, these evaluation metrics are merely appropriate for estimating binary-class imbalance tasks. To extend these metrics to multiclass, some transformations should be considered. *G*-mean computes the geometric mean of all classes' accuracies and is described as follows:

(14) $$\begin{array}{c} G\text{-mean}={\left(\prod_{i=1}^{C}\text{Ac}{\text{c}}_{i}\right)}^{1/C}, \end{array}$$

where Acc_*i*_ denotes the accuracy of the *i*th class. *F*-measure can be transformed as *F*-score [49], which can be calculated by using the following formula:

(15) $$\begin{array}{c} F\text{-score}=\frac{\sum_{i=1}^{C}F\text{-}{\text{measure}}_{i}}{C}, \end{array}$$

where *F*-measure_*i*_ can be calculated further by using the following equation:

(16) $$\begin{array}{c} F\text{-}{\text{measure}}_{i}=\frac{2\times \text{Precisio}{\text{n}}_{i}\times \text{Recal}{\text{l}}_{i}}{\text{Precisio}{\text{n}}_{i}+\text{Recal}{\text{l}}_{i}}, \end{array}$$

and the Acc metric can also be transformed as follows:

(17) $$\begin{array}{c} \text{Acc}=\sum_{i=1}^{C}\left(Acc_{i}\times P_{i}\right), \end{array}$$

where *P*_*i*_ is the percentage of samples in the *i*th class.

To impartially and comprehensively assess the classification performance, we use three extended measures, namely, *G*-mean,*F*-score, and Acc, which are described in (14), (15), and (17), respectively, as evaluation metrics.

We empirically performed threefold cross-validation [16] to evaluate classification performance. Considering the randomness of the sample set partition, each experiment was randomly repeated 10 times. The final values of Acc, *F*-score, and *G*-mean were averaged by these 10 runs. The penalty factor *C* and the width parameter *σ* of RBF kernel function were tuned by using grid search with threefold cross-validation, where *C* ∈ [2^−2^, 2^−1^,…, 2^15^] and  *σ* ∈ [2^−6^, 2^−5^,…, 2^5^]. In addition, the initial dimension of feature space *K* and that of feature subspace *D* are empirically assigned as 100 and 20, respectively. *L*, which indicates the number of base classifiers in each OAA branch, is also empirically assigned as 100.

To demonstrate the advantage of our methods, we evaluated them in comparison with 10 other classification methods, namely, SVM-OAA, SVM-OAO, SVM-DDAG, SVM-ECOC, single SVM-OAA classifier with THR and RUS correction strategies (OAA-SVM(THR) and OAA-SVM(RUS)), ensemble of SVM-OAA without considering class imbalance (EnSVM-OAA), MCSVM [41], Ramp-MCSVM [42], and AdaBoost.NC [19]. To equitably compare the performance of various methods, we used the same common parameters. The other parameters used were the default ones found in references [19, 41, 42].

## 5. Results and Discussions

The experimental results of 12 classification algorithms on 8 datasets are reported in Tables 3, 4 and 5, where the best result in each dataset is highlighted in bold, the second best is underlined, and the worst is italicized. From Tables 3 to 5, we observe the following.

- SVM with various coding strategies exhibits quite similar classification performance in terms of Acc, *F*-score, and *G*-mean evaluation metrics. Compared with its three competitors, SVM-OAA does not show sufficient superiority, although it simplifies transformation by decomposing each multiclass problem to the least binary-class problems. In addition, we found that all four traditional classification algorithms are sensitive to class imbalance.
- Some datasets are sensitive to class imbalance but others are not, as shown by the difference between Acc and *G*-mean values. An Acc value that is much larger than the *G*-mean value means that the corresponding classifier is significantly affected by imbalanced class distribution, which was observed in several datasets used in the study, including Brain_Tumor1, 11_Tumors, and 14_Tumors. Brain_Tumor2 and Lung_Cancer were both slightly sensitive to class imbalance as well. We consider these results to be related to a weighted combination of number of classes, class imbalance ratio, and class overlapping, as explained by previous studies [19, 50, 51].
- Both THR and RUS correction technologies help SVM-OAA classifier promote classification performance on those sensitive datasets. The promotions are better reflected by the *F*-score and *G*-mean metrics, which are used to evaluate the balance level of classification results. Thus, the correction technologies are useless when the classification tasks are robust to class imbalance.
- In contrast with SVM-OAA, the ensemble version EnSVM-OAA helps to slightly improve the overall classification accuracy Acc, with possible sacrifice of two other evaluation metrics on most datasets, which means that classification accuracies between majority and minority classes are further increased.
- Our proposed algorithms outperform other classification algorithms, including several subtle multiclass imbalance classification algorithms [19, 41, 42], in terms of all evaluation criteria for most datasets and especially on the sensitive ones. During the experiments, we observed an interesting phenomenon: EnSVM-OAA(RUS) generally has more stable performance than its partner, although EnSVM-OAA(THR) produces slightly better recognition results on several datasets. We consider that the excessive threshold adjustment negatively affects the recognition accuracy of majority classes to a large extent, which further affects overall prediction accuracy. In practical applications, the decision threshold adjustment function should be subtly designed by considering real distribution of instances.

**Figure 4:**
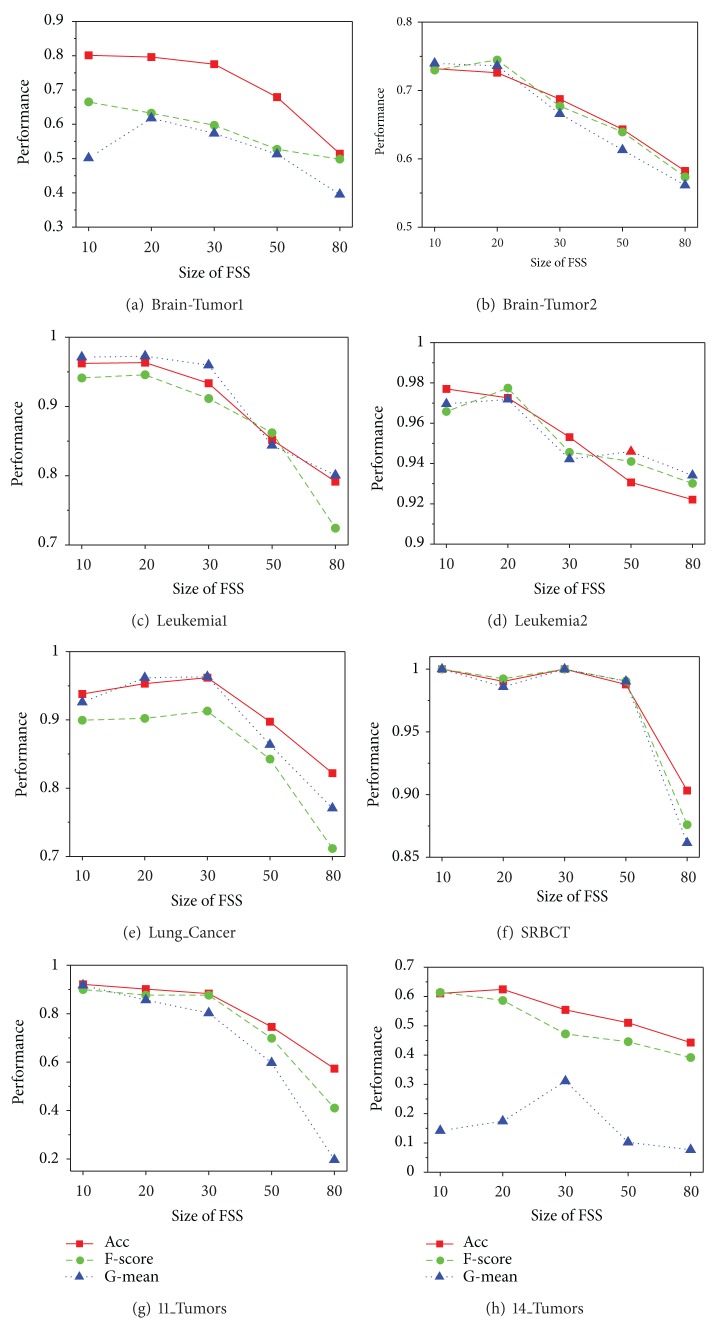
Performance comparison for EnSVM-OAA(THR) algorithm based on different sizes of feature subspace on the eight imbalanced multiclass cancer microarray datasets.

**Figure 5:**
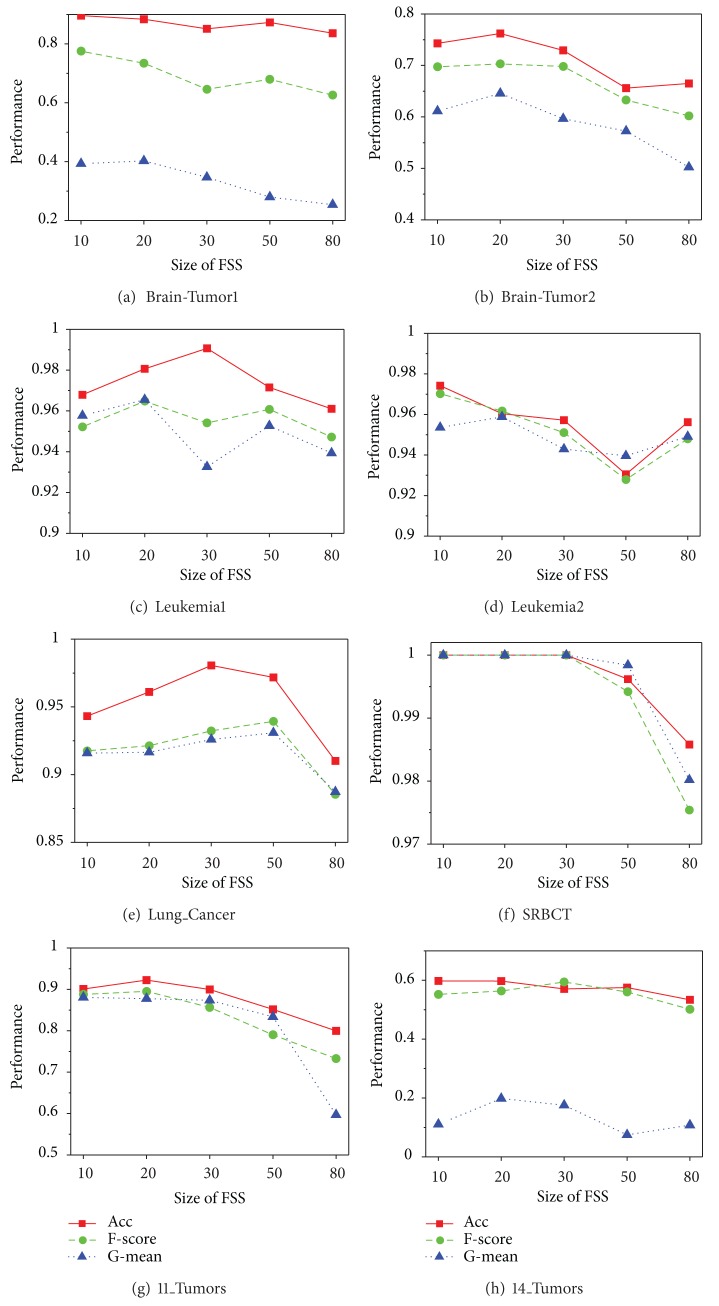
Performance comparison for EnSVM-OAA(RUS) algorithm based on different sizes of feature subspace on the eight imbalanced multiclass cancer microarray datasets.

The classification performance of our proposed algorithms is restricted by many factors, including the size of feature space, the size of feature subspace, and the number of base classifiers; the size of feature subspace is the most significant factor. To clarify its influence mechanism, we designed a group of new experiments in which the dimension of feature subspace is assigned as 10, 20, 30, 50, and 80. The other parameters follow the initial settings in Section 4. The average results of 10 random runs for EnSVM-OAA(THR) and EnSVM-OAA(RUS) are reported in Figures 4 and 5, respectively.

 Although some fluctuations were observed, Figures 4 and 5 nonetheless reveal a common trend that optimal performances often emerge with a feature subspace of 10 to 30 dimensions. With the further increase of the feature subspace dimension, the classification performance drops rapidly, which indicates that selecting a feature subspace with 10 to 30 dimensions can maximize the balanced relationship between accuracy and diversity of base classifiers. This result can be easily explained by the following: extracting a too-small subgroup of feature genes can negatively affect the performance of each base classifier, whereas using too many feature genes can negatively affect diversity among base classifiers. In fact, in practical applications, the optimal dimension can be determined through internal multiple-fold cross-validation of the training sets. The experimental results help guide the construction of the optimal classification model.

## 6. Conclusions

In this paper, we attempted to address multiclass imbalanced classification problem in tumor DNA microarray data by using ensemble learning. The proposed solution contributes in three ways: (1) an improved version of random subspace called feature subspace, which is specifically designed for high-dimensional classification tasks, is proposed to promote a balanced relationship between accuracy and diversity of base classifiers in ensemble learning; (2) two simple correction technologies are adopted in each branch of OAA to alleviate the effect of class imbalance; and (3) a novel ensemble integration strategy called counter voting, which is based on majority voting, is presented to output the final class label. The empirical results show that our proposed classification algorithms outperform many traditional classification approaches and yield more balanced and robust classification results.

Our goal is for the proposed algorithms to be applied in real clinical cancer diagnostic systems based on DNA microarray data in the future. Our future work will consider the extension of correction strategies and classification approaches to deal with this problem and will also explore some efficient solutions with several other coding strategies.

## Figures and Tables

**Pseudocode 1:**
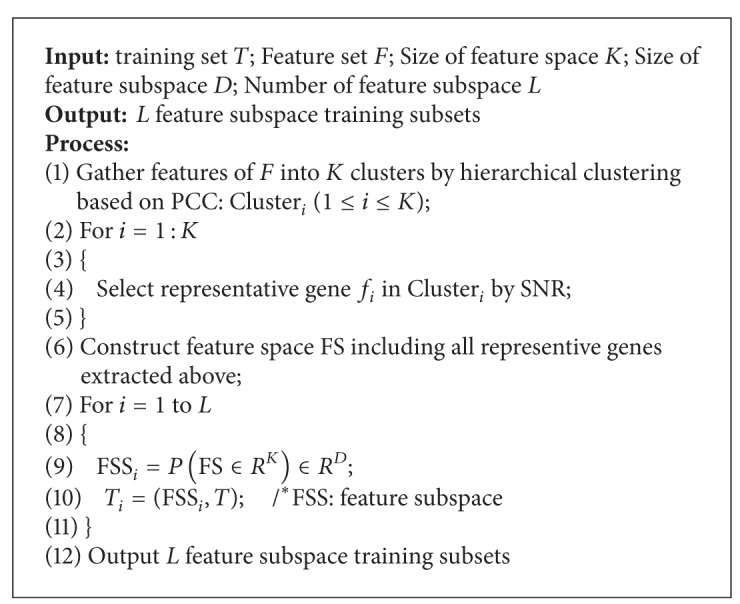
Pseudocode description of the FSS generation algorithm.

**Pseudocode 2:**
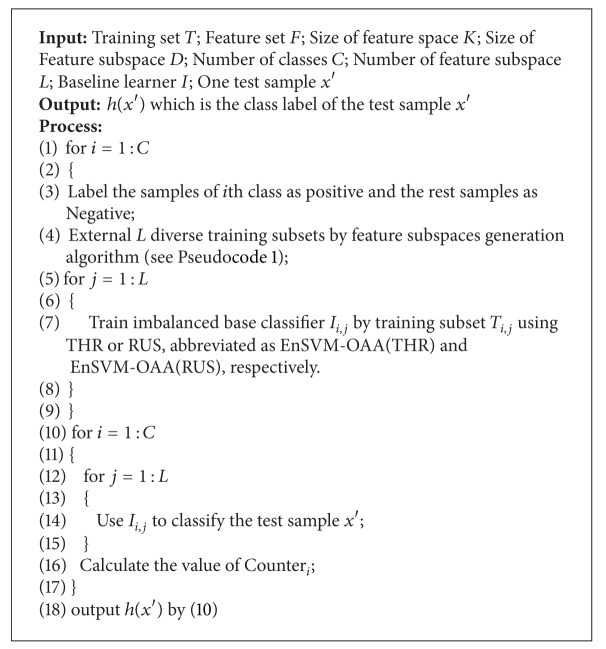
Pseudocode description of the ensemble learning algorithms based on feature subspace and counter voting rule for classifying imbalanced multiclass cancer microarray data.

**Table 1:** Datasets used in this study.

Dataset	Number of samples	Number of classes	Number of genes	Imbalance ratio	Diagnostic task
Brain_Tumor1	90	5	5920	15.00	5 human brain tumor types
Brain_Tumor2	50	4	10367	2.14	4 malignant glioma types
Leukemia1	72	3	5327	4.22	Acute myelogenous leukemia (AML), acute lymphoblastic leukemia (ALL) B-cell, and ALL T-cell
Leukemia2	72	3	11225	1.40	AML, ALL, and mixed-lineage leukemia (MLL)
Lung_Cancer	203	5	12600	23.17	4 lung cancer types and normal tissues
SRBCT	83	4	2308	2.64	Small, round blue cell tumors (SRBCT) of childhood
11_Tumors	174	11	12533	4.50	11 various human tumor types
14_Tumors	308	26	15009	10.00	14 various human tumor types and 12 normal tissue types

**Table 2:** Confusion matrix.

	Predicted positive class	Predicted negative class
Real positive class	True positive (TP)	False negative (FN)
Real negative class	False positive (FP)	True negative (TN)

**Table 3:** Accuracy of various classification methods on eight datasets, where bold represents the best result, underline denotes the second best, and italic labels the worst one in each column, respectively.

Methods	Brain_Tumor1	Brain_Tumor2	Leukemia1	Leukemia2	Lung_Cancer	SRBCT	11_Tumors	14_Tumors
SVM-OAA	0.8596	0.6840	0.9618	0.9334	0.9515	0.9992	0.8932	0.5177
SVM-OAO	0.8611	0.6600	0.9570	0.9369	0.9388	0.9763	0.8851	0.4962
SVM-DDAG	0.8427	0.6760	0.9416	*0.9278 *	*0.8987 *	0.9981	0.8643	*0.4865 *
SVM-ECOC	0.8529	0.6660	0.9558	0.9543	0.9516	0.9916	0.8915	0.5098
SVM-OAA(THR)	*0.7291 *	0.7120	*0.9158 *	0.9621	0.9227	0.9752	0.8862	0.5426
SVM-OAA(RUS)	0.8674	0.7320	0.9596	0.9578	0.9429	0.9988	0.8916	0.5334
EnSVM-OAA	0.8755	0.6980	0.9713	0.9459	0.9571	**1.0000**	0.9021	0.5638
MCSVM	0.8223	*0.6460 *	0.9351	0.9286	0.9315	*0.9628 *	*0.8437 *	0.4988
Ramp-MCSVM	0.8477	0.7420	0.9417	0.9338	0.9296	0.9687	0.9146	0.5012
AdaBoost.NC	0.8516	0.6820	**0.9822**	0.9515	0.9597	**1.0000**	0.8759	0.4928
EnSVM-OAA(THR)	0.7961	0.7260	0.9634	**0.9726**	0.9532	0.9902	0.9017	**0.6246**
EnSVM-OAA(RUS)	**0.8837**	**0.7620**	0.9806	0.9604	**0.9611**	**1.0000**	**0.9224**	0.5974

**Table 4:** *F*-score of various classification methods on eight datasets, where bold represents the best result, underline denotes the second best, and italic labels the worst one in each column, respectively.

Methods	Brain_Tumor1	Brain_Tumor2	Leukemia1	Leukemia2	Lung_Cancer	SRBCT	11_Tumors	14_Tumors
SVM-OAA	0.6524	0.6358	0.9542	0.9328	0.9068	0.9994	0.8468	0.4799
SVM-OAO	0.6732	0.6302	0.9430	0.9315	0.8976	0.9842	0.8322	0.4581
SVM-DDAG	0.6459	0.6420	0.9297	*0.9162 *	0.8762	0.9976	*0.8106 *	*0.4564 *
SVM-ECOC	0.6538	*0.6286 *	0.9418	0.9473	0.9018	0.9902	0.8528	0.4632
SVM-OAA(THR)	*0.6251 *	0.6845	*0.8665 *	0.9602	*0.8621 *	0.9804	0.8453	0.5096
SVM-OAA(RUS)	0.6832	0.6732	0.9352	0.9559	0.9062	0.9992	0.8569	0.5124
EnSVM-OAA	0.6458	0.6437	0.9598	0.9437	0.8975	**1.0000**	0.8664	0.4907
MCSVM	0.6726	*0*.6388	0.9562	0.9306	0.9011	0.9782	0.8229	0.4752
Ramp-MCSVM	0.6918	0.7032	0.9478	0.9375	0.9128	*0.9718 *	0.8776	0.4948
AdaBoost.NC	0.7014	0.6959	**0.9724**	0.9596	**0.9216**	**1.0000**	0.8456	0.4749
EnSVM-OAA(THR)	0.6325	**0.7448**	0.9457	**0.9774**	0.9022	0.9924	0.8768	**0.5869**
EnSVM-OAA(RUS)	**0.7345**	0.7029	0.9648	0.9617	0.9214	**1.0000**	**0.8952**	0.5637

**Table 5:** *G*-mean of various classification methods on eight datasets, where bold represents the best result, underline denotes the second best, and italic labels the worst one in each column, respectively.

Methods	Brain_Tumor1	Brain_Tumor2	Leukemia1	Leukemia2	Lung_Cancer	SRBCT	11_Tumors	14_Tumors
SVM-OAA	0.1012	0.6021	0.9473	0.9354	0.8362	0.9984	0.7981	0.0759
SVM-OAO	*0.0279 *	0.6109	0.9358	0.9253	0.8417	0.9722	0.8042	0.0325
SVM-DDAG	0.1469	0.6128	0.9198	*0.9074 *	*0.8158 *	0.9954	*0.7659 *	0.0468
SVM-ECOC	0.1538	*0.5895 *	0.9436	0.9446	0.8402	0.9946	0.8125	*0.0256 *
SVM-OAA(THR)	0.5754	0.6923	0.9426	0.9658	0.9465	0.9786	0.8143	0.1463
SVM-OAA(RUS)	0.2861	0.6052	0.9369	0.9542	0.8982	0.9994	0.8269	0.1578
EnSVM-OAA	0.0288	0.5963	*0.9194 *	0.9403	0.8540	**1.0000**	0.8284	0.0886
MCSVM	0.4791	0.6281	0.9335	0.9252	0.8876	*0.9688 *	0.8042	0.1059
Ramp-MCSVM	0.5258	0.7288	0.9517	0.9387	0.9012	0.9734	0.8548	0.1472
AdaBoost.NC	0.4326	0.6644	**0.9763**	0.9526	0.9349	**1.0000**	0.8206	0.0652
EnSVM-OAA(THR)	**0.6177**	**0.7362**	0.9727	**0.9718**	**0.9617**	0.9858	0.8562	0.1742
EnSVM-OAA(RUS)	0.4025	0.6457	0.9655	0.9588	0.9165	**1.0000**	**0.8776**	**0.1983**
